# USING INTENSIVE LIFESPAN DATA TO GAIN DEEPER INSIGHT INTO COPING AND EMOTION REGULATION PROCESSES

**DOI:** 10.1093/geroni/igac059.498

**Published:** 2022-12-20

**Authors:** Claire Growney, Jennifer Bellingtier, Carolyn Aldwin

## Abstract

Effective coping and emotion regulation are important for well-being across the lifespan. Successful maintenance or improvement in these processes are often invoked as explanations for age-related stability or enhancement of well-being. In this symposium, we leverage intensive data to gain a deeper understanding of how individuals manage daily emotions and stressors, critically examining evidence for age-related differences and similarities. Growney and English used experience sampling to examine interpersonal emotion regulation in adults aged 25-85, finding a negative association between age and interpersonal emotion regulation strategy use, but evidence suggesting more effective interpersonal emotion regulation in older age. Bellingtier and colleagues present evidence for age similarity in flexible emotion regulation strategy use across hassle domains in an experience sampling study of adolescents and adults aged 14-88, noting that hassle domain differentiation was associated with emotion regulation strategy use. O’Brien and Neupert used daily diaries to examine associations between daily stressor appraisals and affect in adults aged 60-90, identifying daily negative self-views of aging as a moderator which may be particularly consequential in older adulthood. Cerino and colleagues used data from the National Study of Daily Experiences to examine relationships between perceived stressor control and daily affect, highlighting differing findings across domains of interpersonal stressors. Finally, a discussion will center on the value of considering both age-related similarities and differences, age-relevant factors for successful emotion regulation and coping (e.g., negative and positive aspects of social relationships, views of aging), diverse contexts in which these processes occur, and statistical considerations with micro-longitudinal approaches.

